# Immunomodulatory approaches in managing lung inflammation in COVID‐19: A double‐edge sword

**DOI:** 10.1002/iid3.1020

**Published:** 2023-09-26

**Authors:** Geetika Verma, Manish Dhawan, AbdulRahman A. Saied, Geetika Kaur, Reetesh Kumar, Talha Bin Emran

**Affiliations:** ^1^ Department of Experimental Medicine and Biotechnology Post Graduate Institute of Medical Education and Research (PGIMER) Chandigarh India; ^2^ Department of Microbiology Punjab Agricultural University Ludhiana India; ^3^ Trafford College Altrincham UK; ^4^ Ministry of Tourism and Antiquities, Aswan Office Aswan Egypt; ^5^ Department of Opthalmology, Visual and Anatomical Sciences Wayne State University School of Medicine Detroit Michigan USA; ^6^ Department of Agricultural Sciences, Institute of Applied Sciences and Humanities GLA University Mathura India; ^7^ Department of Pharmacy, Faculty of Allied Health Sciences Daffodil International University Dhaka Bangladesh; ^8^ Department of Pathology and Laboratory Medicine, Warren Alpert Medical School & Legorreta Cancer Center Brown University Providence Rhode Island United States

**Keywords:** COVID‐19, inflammation, lung, SARS‐CoV‐2, therapeutics

## Abstract

**Introduction:**

The novel coronavirus infectious disease 2019 (COVID‐19) which is caused by severe acute respiratory syndrome coronavirus 2 (SARS‐CoV‐2) has emerged as a gigantic problem. The lung is the major target organ of SARS‐CoV‐2 and some of its variants like Delta and Omicron variant adapted in such a way that these variants can significantly damage this vital organ of the body. These variants raised a few eyebrows as the outbreaks have been seen in the vaccinated population. Patients develop severe respiratory illnesses which eventually prove fatal unless treated early.

**Main Body:**

Studies have shown that SARS‐CoV‐2 causes the release of pro‐inflammatory cytokines such as interleukin (IL)‐6, IL‐1β and tumor necrosis factor (TNF)‐α which are mediators of lung inflammation, lung damage, fever, and fibrosis. Additionally, various chemokines have been found to play an important role in the disease progression. A plethora of pro‐inflammatory cytokines “cytokine storm” has been observed in severe cases of SARS‐CoV‐2 infection leading to acute respiratory distress syndrome (ARDS) and pneumonia that may prove fatal. To counteract cytokine storm‐inducing lung inflammation, several promising immunomodulatory approaches are being investigated in numerous clinical trials. However, the benefits of using these strategies should outweigh the risks involved as the use of certain immunosuppressive approaches might lead the host susceptible to secondary bacterial infections.

**Conclusion:**

The present review discusses promising immunomodulatory approaches to manage lung inflammation in COVID‐19 cases which may serve as potential therapeutic options in the future and may prove lifesaving.

## Highlights

- COVID‐19 mainly affects the lungs and in severe cases may lead to acute respiratory distress syndrome (ARDS) and pneumonia.
- Surprisingly, COVID‐19 can affect even the vaccinated population suggesting immune escape of the SARS‐CoV‐2 variants.
- Immunomodulatory approaches may modulate the immune response reducing lung inflammation and lessen the chances of an infected person developing ARDS or pneumonia.
- However, these approaches should be employed wisely based on the severity of the disease and where the benefits are greater than the risk involved.

## Graphical abstract

The novel coronavirus infectious disease (COVID‐19) mainly affects the lungs and in severe cases may lead to acute respiratory distress syndrome (ARDS) and pneumonia. • Immunomodulatory strategies may reduce lung inflammation and lessen the chances of an infected person developing ARDS or pneumonia.

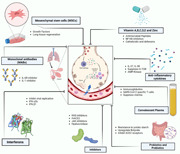

## 1 INTRODUCTION

The most contagious coronavirus disease 2019 (COVID‐19) is caused by severe acute respiratory syndrome coronavirus 2 (SARS‐CoV‐2) in humans.^1^ It has not been previously identified in humans, hence its name “novel” coronavirus. Since it is novel, therefore the whole human population around the globe has been susceptible to SARS‐CoV‐2, which made this a matter of serious concern.^2^ The problem of COVID‐19 has worsened because of its capacity to transmit at a very rapid pace from one person to another.

The most usual symptoms of COVID‐19 include fever, cough, shortness of breath and fatigue; and severe cases of COVID‐19 show pneumonia, sepsis, acute respiratory distress syndrome (ARDS)^3^, ^4^ and septic shock, all potentially leading to death.^5^, ^6^ Huang and colleagues reported that about 80% of infections are mild or asymptomatic, 15% are severe which require oxygen and 5% are critical infections which require ventilation.^7^ Patients are treated using a symptomatic approach and by providing supportive care like oxygen therapy, fluid management that proves to be beneficial in some cases. For severely ill cases, a combination of different pharmaceutical drugs is being employed but the use of these drugs needs to be assessed carefully. The use of corticosteroids to minimize hyperinflammation via immunosuppression in critically ill patients might not be advisable as such immunosuppression may impair induction of type‐I interferon (antiviral) responses to COVID‐19.^8^ Therefore, designing other safe immunomodulatory strategies are need of the hour.

Vaccines against SARS‐CoV‐2 are also being employed to prevent COVID‐19 and are proven efficacious. However, their efficacy against changing mutants of SARS‐CoV‐2 need to be ascertained. Furthermore, the duration of protection imparted by a vaccine is yet to be determined. The emergence of highly transmissible variant(s) of concern (VoC) of SARS‐CoV‐2, such as Delta and Omicron, owing to mutations in the gene that encodes for spike glycoprotein has raised serious concerns about the effectiveness of available vaccines as the outbreaks have been seen in the vaccinated population.^9^, ^10^ Additionally, the effectiveness of majorly available monoclonal antibodies (MABs) against the highly mutated variant that is, Omicron raised a need to develop the alternative medications to treat COVID‐19.^11^ Adverse events like cytokine storm have been observed following receipt of second dose of COVID‐19 vaccination in four cases, showing immune dysregulation might have occurred,^12^ suggesting further the need of immunomodulatory approaches (Table 1).

**Table 1:** Showing list of various immunomodulatory approaches employed for treating lung inflammation in COVID‐19 patients.

S. No.	Immunomodulatory approach	Mechanism/Pathway/Cells involved	Advantages	Disadvantages
1.	Interferon immunotherapy	✓ IFNβ1 up regulates the expression of CD73^46^ ✓ production of anti‐inflammatory agent like adenosine^46^	Anti‐inflammatory and antiviral properties^46^, ^47^	Flu, hematological toxicity, enhanced transaminases, psychiatric sequelae, nausea, and fatigue^49^
2.	Monoclonal antibodies	✓ Blocking the interleukin receptors like IL‐1 and IL‐6^51^, ^55^	Inhibits inflammation	Hypersensitivity, hepatitis, malignancies, gastrointestinal perforations, and cardiovascular risk^60^
3.	Anti‐inflammatory cytokine	✓ IL‐38 blocks the pro‐inflammatory cytokines like IL‐1β^64^ ✓ IL‐37 acts through mammalian target of rapamycin (mTOR) and increases the adenosine monophosphate (AMP) kinase^62^	IL‐37 suppresses innate and adaptive immune response and inhibits lung inflammation^61^	It may affect the antiviral immune response
4.	Janus kinase (JAK) inhibition	✓ Baricitinib (Olumiant), act against JAK1 and JAK2, a family of intracellular, non‐receptor tyrosine kinases^68^ ✓ Ruxolitinib diminish the cytotoxic T lymphocytes and up regulating the regulatory T (Treg) cells^30^	Baricitinib interrupts the virus entry into target cells and inhibits cytokine release^68^ Ruxolitinib works as an immunomodulator^30^	Broad immunosuppression^70^, ^71^
5.	Convalescent plasma	✓ Action of antibodies present in plasma that suppress the infection^76^, ^77^, ^78^	Passive immunity from donor to recipient	Transmission of the potential pathogen^80^ Antibody‐dependent infection enhancement^81^
6.	Mesenchymal stem cells (MSCs)	✓ Immunomodulatory and differentiation properties of MSCs can prevent lung damage by regeneration^82^, ^83^, ^84^, ^85^	Repair the damaged tissue by producing growth factors^80^, ^81^	Pre‐activation of MSCs is required^86^ Side‐effects include headache, facial flushing, allergic rash, and fever^84^
7.	Vitamin supplementation	✓ Vitamin B3 inhibits infiltration of neutrophils into the lungs^89^	Exhibits anti‐inflammatory effects^83^, ^84^	Vitamin B3 paradoxically lead to the development of significant hypoxemia^88^
8.	Probiotics	✓ *Lactobacillus casei* reduce TLR4 expression^95^ ✓ Lipopeptides derived from the probiotics inactivates spike protein of SARS‐CoV‐2 and ACE2^98^	Exhibits anti‐inflammatory effectsStrengthen lung immunity	Dose of the probiotic May cause mild gastrointestinal side effects
9.	Prebiotics	✓ Inhibit ACE2 receptor expression^107^	May inhibit lung inflammation	Dose of the prebiotic
10.	Camelid‐derived nanobodies	✓ Detect novel antigenic sites and penetrate tissues deeply^108^	High specificity, chemo‐ and thermostability, solubility, reduced sensitivity to steric hindrances^108^, ^109^	High Cost of production Immune suppression

COVID‐19 infection triggers destructive immune hyper reaction via several pathways. One such is the Toll‐Like Receptor (TLR) activation leading to a cytokine storm which is characterized by an overproduction of inflammatory factors and cytokines.^13^, ^14^, ^15^, ^16^ TLRs play a central role in host responses to microbial molecules in lung and various other organs.^17^, ^18^, ^19^, ^20^ This hyper‐inflammation imposes significant burden and even inhibit the patient's body to generate adequate number of antibodies against SARS‐CoV‐2.^21^ This cytokine storm is the reason underlying viral sepsis and lung inflammation induced injury, pneumonitis, ARDS, respiratory failure, shock, organ failure, and death.^14^, ^15^, ^16^, ^22^ Therefore, it is important to develop strategies which can counteract or neutralize this cytokine storm by modulating immune response and hence inhibit lung inflammation and damage, inhibit viral replication, and promote viral clearance; thus, eventually saving a life.

Additionally, the high rate of malnourishment among the elderly who are hospitalized is a significant possible contributing factor in the poor prognosis of the COVID‐19 disease. Nutritional approaches to enhance the immune system's optimum function are often overlooked in public health discourses on COVID‐19 prevention and treatment. Given the importance of macro‐ and micronutrients in immune function, they must be included in COVID‐19 therapeutic interventions. Micronutrient deficiencies should thus be treated as soon as possible, particularly in the elderly and other susceptible populations since suboptimal status or deficits in certain immune‐relevant micronutrients lower the resilience to viral infection and impair the immunological response.^23^ Keeping such scenario in view, the present review highlights the various aspects of recent promising strategies which are being adopted to potentially inhibit lung inflammation, injury or damage and promote lung regeneration and repair in COVID‐19 treatment. These immunomodulating strategies are employed alone or in combination with other antiviral drugs/agents to serve as effective therapeutic option. Several clinical trials using these strategies are being carried out which may be approved in the long run to treat pulmonary inflammation in COVID‐19 once positive results are seen. The information is vital for the researchers and readers to explore new dimensions in managing lung inflammation in COVID‐19 patients.

## 2 HOW SARS‐COV‐2 ENTERS LUNG AND CAUSE INFECTION?

**Figure 1:**
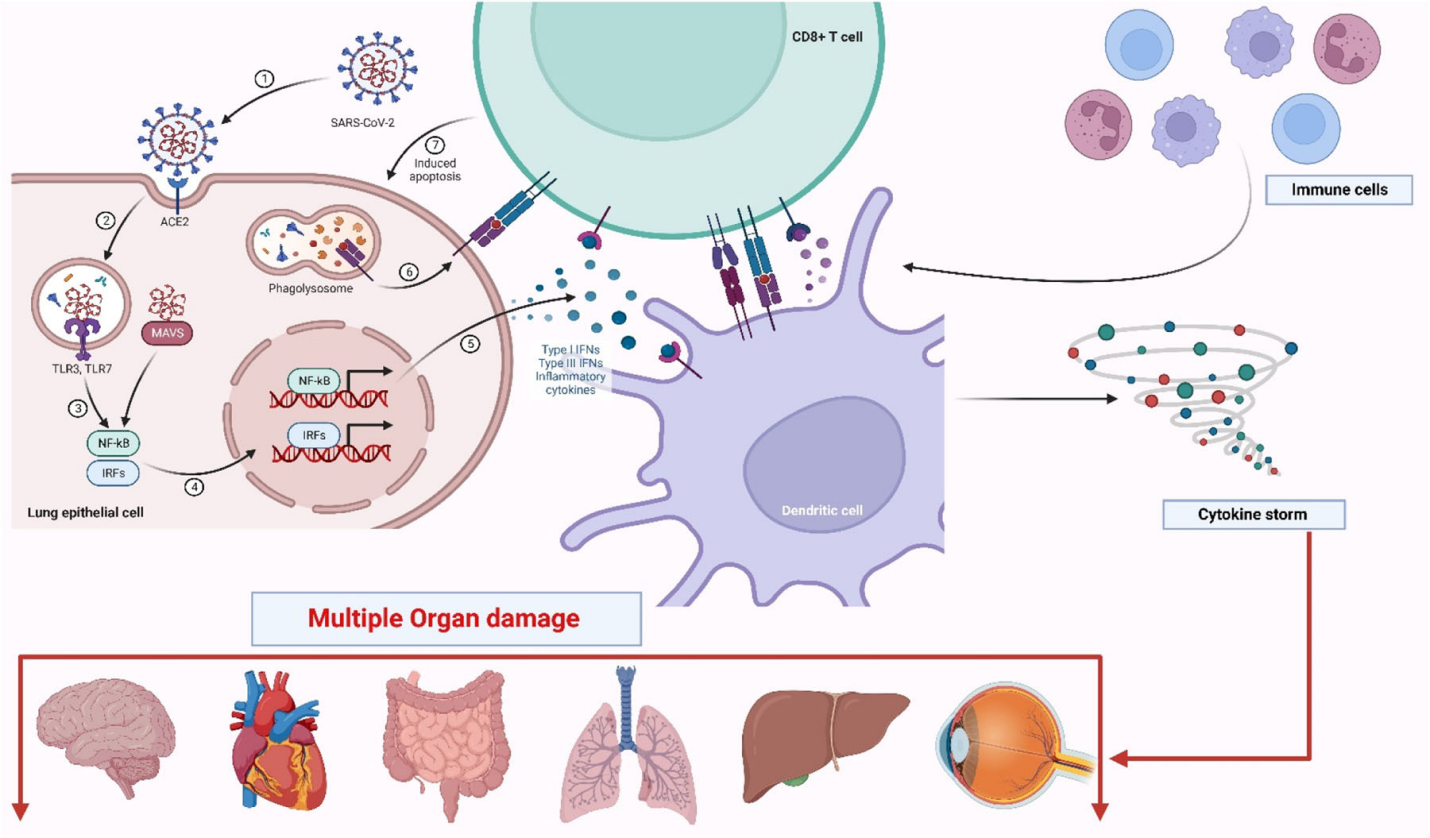
Schematic representation of the infection mechanism of SARS‐CoV‐2 and the generation of the immune response against the viral infection. The entry of SARS‐CoV‐2 virus is mediated by ACE2 receptor (1) on lung epithelial cell surface followed by activation of TLR3 and TLR7 (2) which further activates NF‐kB and other pathways (3) to secrete Type I IFNs and a plethora of cytokines leading to cytokine storm (4, 5). These cytokines exert their effect after binding to various receptors on T cells and other antigen presenting cells like dendritic cells (DCs) which again lead to generation of more cytokines and chemokines. It is interesting to note that the excessive release of inflammatory cytokines known as cytokine storm can lead to the severe damage of the lungs. Additionally, this cytokine storm has been considered as the primary reason of multiple organ damage and death in the severely infected patients with COVID‐19 (The figure was created with BioRender.com).

SARS‐CoV‐2 finds its way in human alveolar cells via Angiotensin‐converting enzyme 2 (ACE2), as the C‐terminal domain of spike protein of SARS‐CoV‐2 has higher affinity for human ACE2 receptor.^24^, ^25^, ^26^ Therefore, human organs like lung (alveolar epithelial cells) and small intestine (enterocytes) with high level of ACE2 expression are the potential target of SARS‐CoV‐2.^25^, ^27^ The high expression of the ACE2 receptor in elderly patients also correlates with high viral load.^27^ SARS‐CoV‐2 causes activation of several signaling pathways like nuclear factor κB (NF‐κB) and subsequently the release of pro‐inflammatory cytokines like pro‐IL‐1β which is then cleaved by caspase‐1, followed by inflammasome activation, leading to the production of active interleukin (IL)‐1β, a mediator of lung inflammation, fever and fibrosis^14^, ^28^, ^29^ (Figure 1). Severe cases of SARS‐CoV‐2 infection are associated with cytokine storm or cytokine release syndrome where cytokines like IL‐6, IL‐1β, tumor necrosis factor (TNF)‐α, monocyte chemoattractant protein (MCP)‐1, macrophage inflammatory protein (MIP)‐1A, Granulocyte colony‐stimulating factor (G‐CSF), IL‐10, IL‐7, IL‐2 and interferon‐γ‐inducible protein (IP)‐10 may induce lung inflammation and damage; hence causing severe respiratory illness.^14^, ^30^ Further, COVID‐19 disease severity has also been linked to the reduction in the regulatory T cells (Tregs) that exhibits immunoregulatory and immunosuppressive properties.^31^

## 3 IMMUNOMODULATORY STRATEGIES: NEED OF THE HOUR

**Figure 2:**
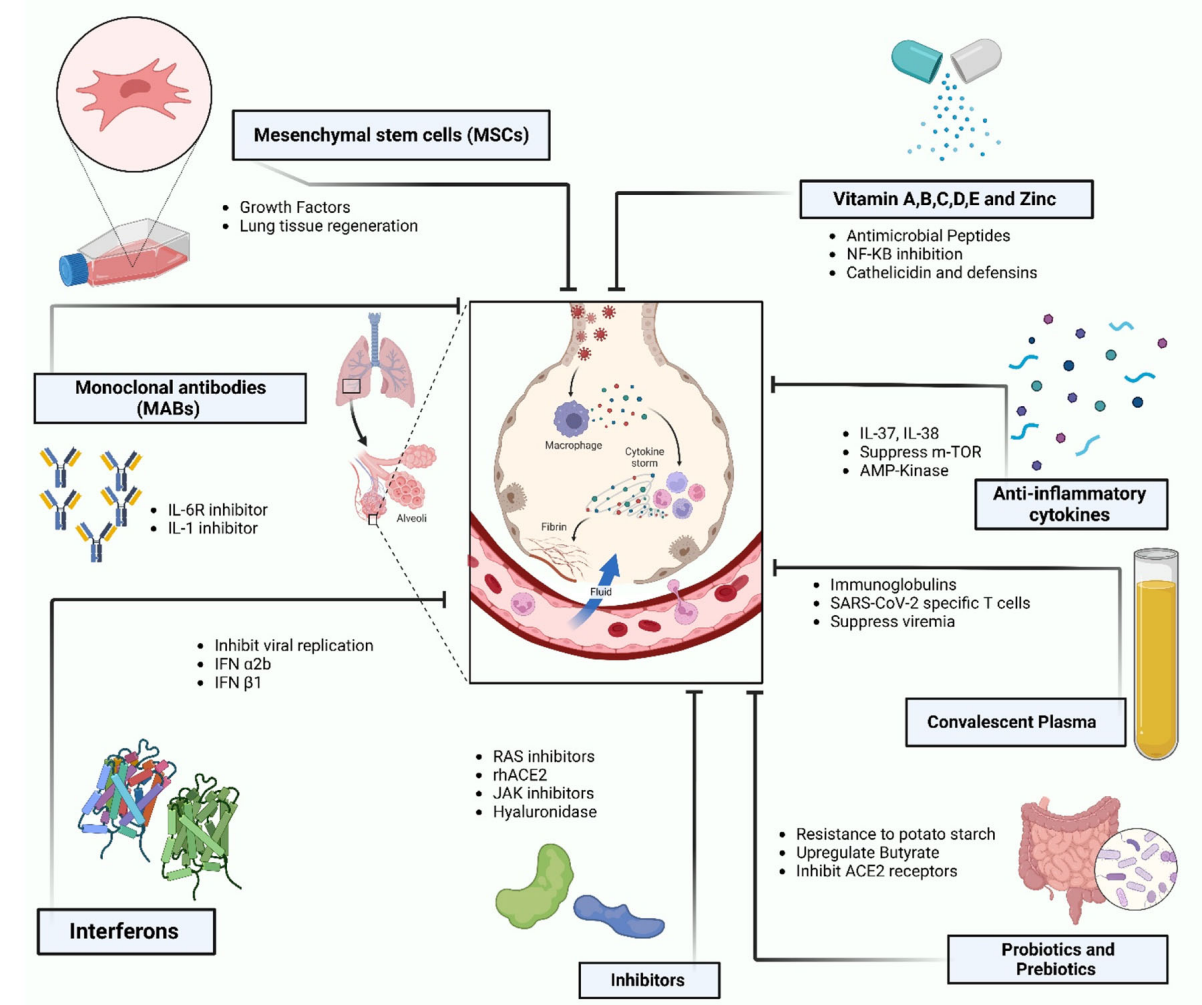
Various immunomodulatory strategies to treat lung inflammation in COVID‐19. The entry of SARS‐CoV‐2 virus in lungs can cause cytokine storm which could be inhibited by strategies as listed. ACE: Angiotensin‐converting enzyme; AMP: adenosine monophosphate; IFNs: Interferons; IL: Interleukin; JAK: Janus kinase; MABs: Monoclonal Antibodies; mTOR: Mammalian Target of Rapamycin; NF: Nuclear Factor; RAS: Renin‐angiotensin system; rhACE: Recombinant human angiotensin‐converting enzyme; SARS‐CoV‐2: severe acute respiratory syndrome coronavirus 2; Mesenchymal stem cells (The figure was created with BioRender.com).

Lung is the major organ targeted in COVID‐19 infection. Thus, to minimize lung inflammation and damage, several clinical trials are being performed using various immunomodulatory strategies which are discussed below (Figure 2).

### 3.1 Interferon immunotherapy

Interferons (IFNs) are the signaling antiviral glycoproteins released by a virus‐infected cell to protect nearby cells. These can be broadly classified into three major groups viz. Type I, Type II and Type III IFNs depending upon the structure of cell membrane receptor.^32^ Since, these molecules have the capacity to modulate the immune system, hence may serve as feasible therapeutic option against COVID‐19 infections.

Viruses are evolving at a rapid pace and have developed novel mechanisms to neutralize or suppress the induction of IFNs in host to establish infection. Similarly, coronavirus infection suppresses type I IFN response, by targeting the signaling leading to type I IFN production, which correlates with disease severity.^33^ Recent study by Zheng and colleagues has shown that SARS‐CoV‐2 membrane protein inhibits type I and III IFN production by targeting RIG‐I/MDA‐5 signaling.^34^ Purified fractions of IFNs if administered exogenously interferes with viral replication and prevent its spread by several mechanisms like slowdown of cell metabolism or secretion of cytokines promoting the activation of the adaptive immunity.^35^, ^36^ Type I IFNs have been investigated to treat the deadly SARS‐CoV‐2.^37^, ^38^ The potential role of IFNα, IFNβ and IFNλ is discussed as follows.

#### 3.1.1 Interferon (IFN)‐α

In China, the use of IFNα by vapor inhalation, in combination with ribavirin in COVID‐19‐infected patients, has been recommended.^39^, ^40^ The vapor inhalation is advantageous in targeting, specifically the respiratory tract. It was shown that IFNα2b sprays can reduce the infection rate of SARS‐CoV‐2.^41^ A study shows that IFN‐I can be used as a prophylaxis against SARS‐CoV‐2, which is confirmed by the in vitro efficacy of interferon pretreatment against the virus.^42^ Several clinical trials are aimed at evaluating the efficacy of combination of drugs (lopinavir/ritonavir) and IFNα2b for the treatment of COVID‐19. IFN‐α has also shown promising adjuvant properties in human and animal studies.^43^ Therefore, it might be used as a potential candidate as an adjuvant for COVID‐19 vaccine after suitable analysis and trials.

#### 3.1.2 Interferon (IFN)‐β

IFNβ1 seems to be the most appropriate interferon to treat COVID‐19 infection as it provides protective immunity to lung.^44^ IFNβ1 upregulates the expression of cluster of differentiation 73 (CD73) in pulmonary endothelial cells which leads to the production of adenosine, an endogenous anti‐inflammatory agent, and helps in the maintenance of endothelial barrier function.^45^ Clinical trials (NCT04343768) using combination of lopinavir/ritonavir with ribavirin and IFNβ1b administered subcutaneously for the treatment of COVID‐19 proved beneficial. IFNβ1 serves as a safe and easy to upscale treatment option against COVID‐19 in the early stages of infection.^46^

#### 3.1.3 Interferon (IFN)‐λ

IFN‐λ, type III IFNs, are also antiviral like type I IFNs^46^, ^47^; however, their receptors are expressed on a limited subset of host cells. Hence, treatment with IFN‐λ may be associated with few systemic adverse effects.^46^ Clinical trials (NCT04354259) evaluating the efficacy and safety of peg‐interferonλ‐1a for treatment of COVID‐19 are expected to fetch positive results.

#### 3.1.4 Concerns

Detrimental effects of using IFNα include flu, hematological toxicity, enhanced transaminases, psychiatric sequelae, nausea, and fatigue.^48^ Therefore, patients likely to benefit from IFN treatment should be identified. The timing of IFNs administration needs to be assessed as positive effects were seen when IFN‐I was administered shortly after infection, but it failed to inhibit viral replication and had side‐effects when administered later.^49^ Many clinical trials are ongoing with the type I interferons and few with type III IFNs for COVID‐19.

### 3.2 Cytokine specific blockade

Pro‐inflammatory cytokines can be blocked by cytokine inhibitors, antagonists, and monoclonal antibodies (MABs) rendering them no longer cause inflammation. MABs have been directed against various molecules like granulocyte monocyte colony stimulating factor (GM‐CSF), IFN‐γ, IL‐1β, chemokines etc. to fight against COVID‐19, such as Mavrilimumab (formerly CAM‐3001). However, clinical trials are mainly going using MABs against IL‐1 and IL‐6 as major targets.

#### 3.2.1 MABs against interleukin 1 (IL‐1)

Shakoory et al. reported that blocking IL‐1 receptor with anakinra (recombinant interleukin‐1 receptor antagonist) in sepsis, showed significant survival in patients with hyperinflammation, without risk of side effects.^50^ IL‐1 is up regulated in COVID‐19 patients—one of the culprits in lung inflammation and damage.

#### 3.2.2 MABs against interleukin 6 (IL‐6)

IL‐6 blood measurements may serve as a diagnostic marker in severe COVID‐19 cases.^51^, ^52^, ^53^ COVID‐19 induces elevated levels of IL‐6 for at least 2 weeks after onset of the disease and besides IL‐6, molecules associated with IL‐6 pathway, such as ADAM metallopeptidase domain 17 (ADAM‐17), SARS‐CoV ssRNA, dual‐specificity phosphatase 1 (DUSP1) and p38 mitogen‐activated protein kinase (MAPK) have been suggested as therapeutic targets.^54^ MABs against IL‐6 receptor include Tocilizumab (Actemra), Siltuximab (Sylvant), Sarilumab (Kevzara) have been employed as treatments in several clinical trials to treat COVID‐19. A multicentre, randomized controlled trial of tocilizumab (ChiCTR2000029765) has been approved in China for COVID‐19 pneumonia patients with elevated IL‐6.^14^ In contrast, tocilizumab treatment was found to have no statistically significant effect on mortality reduction in hospitalized patients with severe SARS‐CoV‐2 infection. Additionally, it was determined that using tocilizumab had minimal impact on oxygen needs or clinical advancement.^55^ There is insufficient data about the efficiency and safety of tocilizumab, according to a recent literature assessment of multiple clinical trials, including the use of tocilizumab to treat COVID‐19. As a result, it is necessary to examine and then modify tocilizumab's therapeutic usage.^56^, ^57^, ^58^

#### 3.2.3 Concerns

IL‐6 suppression may increase the risk of hypersensitivity, hepatitis, malignancies, gastrointestinal perforations, and cardiovascular risk.^59^

### 3.3 Inhibition of pro‐inflammatory cytokines: Anti‐inflammatory cytokine approach

The inhibition of pro‐inflammatory cytokines by anti‐inflammatory cytokines (IL‐37, IL‐38) may provide a new therapeutic dimension. IL‐37 suppresses innate and adaptive immune response^60^ through mammalian target of rapamycin (mTOR), increases the adenosine monophosphate (AMP) kinase and inhibits lung inflammation via IL‐18Rα receptor,^61^ thereby, providing relief in both lung inflammation and fever.^14^

Another inhibitory cytokine, IL‐38 is the most recent cytokine of the IL‐1 family, produced by B cells and macrophages.^62^ IL‐38 is more potent than IL‐37 and a suppressor cytokine which inhibits IL‐1β and other pro‐inflammatory IL‐family members.^63^ IL‐38 is a potential therapeutic cytokine which inhibits inflammation in viral infections, including that caused by COVID‐19, providing a new relevant strategy.^14^

#### 3.3.1 Concerns

Immunosuppression in critically ill patients needs to be assessed carefully as it may affect the antiviral immune response.

### 3.4 Renin‐angiotensin system (RAS) inhibitors: A controversial approach

SARS‐CoV‐2 binds to angiotensin converting enzyme 2 (ACE2) protein, which is an important component of renin‐angiotensin system (RAS).^64^ ACE2 is an enzyme that inhibits lung inflammation via RAS and increased ACE2 activity seems to improve acute respiratory distress syndrome (ARDS).^65^ Inhibition of RAS by angiotensin II receptor blockers (ARB) or angiotensin converting enzyme inhibitors (ACEi) leads to up regulation of ACE2 and hence, attenuation of SARS‐CoV‐induced ARDS.^66^ This is highly important, because most deaths in COVID‐19 infection occur due to ARDS. Thus, ACE inhibitors and ARBs might alleviate the pulmonary manifestations in COVID‐19.

#### 3.4.1 Concerns

ACE2 upregulation by RAS inhibitors will increase the cellular access points for SARS‐CoV‐2 entry and might enhance the progression of COVID‐19. These multiplied viral entry points might be the reason behind the high morbidity and mortality in COVID‐19 patients with hypertension or diabetes because patients having hypertension and/or diabetes take drugs targeting the renin‐angiotensin system (RAS) to lower blood pressure and protect kidney. Therefore, the role of RAS in COVID‐19 should be exploited further.

### 3.5 Recombinant human angiotensin‐converting enzyme 2 (rhACE2)

Recombinant, soluble glycosylated form of the human angiotensin‐converting enzyme 2 (rhACE2, APN01) has the potential to block SARS‐CoV‐2 infection and reduce lung damage. APN01 is the first drug approved to treat COVID‐19 that specifically targets SARS‐CoV‐2. APN01 was developed by APEIRON biologics to treat acute lung injury (ALI), ARDS and pulmonary arterial hypertension. rhACE2 mimics the human enzyme ACE2, which is used by the virus for entry into the host cells. The virus binds to soluble rhACE2, instead of ACE2 on the cell surface; hence, no longer infect the host cells. APN01 decrease levels of its target peptide angiotensin II, with a tendency to lower plasma IL‐6 concentrations.^65^ Therefore, rhACE2 reduces the inflammatory reactions in the lungs and protects against ALI or ARDS.

#### 3.5.1 Concerns

The development of ACE2 target therapies appears promising although theoretically limited only to selected patients. It is important to establish therapy in patients who meet ARDS criteria without concomitant hemodynamic instability.

### 3.6 Janus kinase (JAK) inhibition

Baricitinib (Olumiant), a JAK inhibitor, act against JAK1 and JAK2, a family of intracellular, non‐receptor tyrosine kinases, interrupting the virus entry into target cells and inhibits cytokine release.^67^ This valuable action of baricitinib forms the rationale to perform clinical trials on COVID‐19 patients. Since, it does not interact with antivirals it may be used in combination. Baricitinib has also been approved for the treatment of rheumatoid arthritis.^68^ Another inhibitor used is Ruxolitinib, an inhibitor of JAK 1/2. Ruxolitinib works as an immunomodulator diminishing the cytotoxic T lymphocytes and upregulating Treg cells.^29^

#### 3.6.1 Concerns

Broad immunosuppression in COVID‐19 patients using JAK inhibitors might also block the antiviral immune response as JAK‐STAT (Signal Transducer and Activator of Transcription) signaling is a major component of the type‐I interferon pathway.^69^ Tofacitinib, a JAK inhibitor, has been shown to inhibit interferon‐α production in vitro.^70^ Suppression of interferon or other mediators could also promote secondary bacterial infection and further complicate the disease course.

### 3.7 Inhibiting hyaluronan: Immunomodulation using natural products

The inflammatory cytokines like IL‐1 and TNFα are strong inducers of hyaluronan‐synthase‐2 (HAS2), which secrete hyaluronan in lung alveolar epithelial cells and fibroblasts.^71^ Hyaluronan causes difficulty in breathing as it can absorb water up to 1000 times its molecular weight. Therefore, inhibiting the production of hyaluronan helps treating breathing problem in ARDS and COVID‐19 patients. For this purpose, hyaluronidase and hymecromone (4‐Methylumbelliferone, 4‐MU) which is clinically approved bile therapy drug and an inhibitor of HAS2^72^ are used. Hyaluronidase has immunomodulating properties and suppresses neutrophil infiltration and cytokine production.^73^ Traditional Chinese medicine consists of 4‐MU, explaining the observed efficacy of the herbal medicine in some COVID‐19 patients in China.^74^

#### 3.7.1 Concern

Dosing and time of administration needs to be assessed carefully for effectiveness.

### 3.8 Convalescent plasma

Identifying the patients with cytokine storm and administering them with convalescent plasma isolated from COVID‐19 recovered donors may prove to be a life‐saving therapeutic option. Convalescent plasma from healthy donors who recovered from SARS CoV‐2 can reduce the cytokine storm and to replenish the patient's own antibodies in the acutely infected phase of the COVID‐19. Convalescent plasma or immunoglobulins is being used as a last alternative to improve the survival rate of COVID‐19 patients.^75^ Several studies showed lower mortality rates in patients treated with convalescent plasma.^75^, ^76^, ^77^ This might be due to the action of antibodies present in plasma that suppress the infection. Viremia usually peaks in the first week of most viral infections and a primary immune response is developed by 10–14 days which is followed by virus clearance.^75^ Therefore, convalescent plasma treatment to be effective, it should be administered at the early stage of COVID‐19 infection. Passive immunity can be acquired by adoptive transfer of SARS‐CoV‐2 specific T cells from convalescent donors into newly infected patients. Clinical trial [NCT04351659] is going on for preparing SARS‐CoV‐2 specific T cells from convalescent donors for urgent clinical use.

#### 3.8.1 Concerns

The effect of other combination treatments on the relationship between convalescent plasma and antibody level, including antiviral drugs, steroids, and immunoglobulin should be evaluated.^78^ One of the risks associated with plasma transfusion is the transmission of the potential pathogen for which methylene blue photochemistry is useful to inactivate the residual virus and to maintain the activity of neutralizing antibodies.^79^ Another rare risk associated is antibody‐dependent infection enhancement at sub neutralizing concentrations which may inhibit innate immunity and allow virus to grow intracellularly.^80^

### 3.9 Use of mesenchymal stem cells

This strategy has been adopted by China recently, but the results are awaited. Mesenchymal stem cells (MSCs) based treatment seems promising therapeutic approach and several clinical trials are ongoing. MSCs can reduce the macrophages, neutrophils, and DCs infiltration into the lungs and levels of various cytokines involved in inflammation.^81^, ^82^ The immunomodulatory and differentiation properties of MSCs can prevent lung damage by regeneration; repair the damaged tissue by producing growth factors and block inflammation by counteracting the cytokine storm.^81^, ^82^, ^83^ MSCs secrete paracrine factors that can help in restoring pulmonary epithelial and endothelial lining and minimize the pulmonary edema, eventually rebuilding the lung cell environment and structure.^83^, ^84^ Human umbilical cord‐derived MSCs (UC‐MSCs) are also being employed in clinical trials to treat COVID‐19. These offer unique advantages over MSCs as they are easy to collect, less immunogenic, free from ethical controversies and have rapid proliferation rate.

#### 3.9.1 Concerns

It is worth mentioning that MSCs need to be activated prior by IFNγ to exhibit anti‐inflammatory effects.^74^ Wang and colleagues suggested pretreatment of MSCs with IFNγ or TNFα or IL‐1 could be more effective in tissue repair and suppression of hyperactive immune response.^85^ Other concerns regarding isolation of MSCs, the cost and speed of the whole process needs to be considered. Transient side‐effects like headache, facial flushing, allergic rash, and fever have been reported after MSC infusion which generally disappear within 24 h.^83^

### 3.10 Vitamin supplementation

An ongoing clinical trial [NCT04323228] at King Khalid University Hospital aims at evaluating the potential of specific nutrients such as omega‐3 fatty acid and vitamins (Vitamin A, C, E) in modulating the host immune response and remodeling the cytokine storm associated with COVID‐19. Other vitamins explored for therapeutic uses in COVID‐19 are discussed.

#### 3.10.1 Vitamin B3

Vitamin B3 (niacin or nicotinamide) is beneficial in preventing lung injury or damage by attenuating the production of pro‐inflammatory cytokines particularly TNF‐α, IL‐6 and IL‐1β through NF‐κB inhibition.^86^, ^87^ It significantly inhibits infiltration of neutrophils into the lungs and exhibit potent anti‐inflammatory effects.^88^ Therefore, supplementing Vitamin B3 in diet of COVID‐19 patients may prove valuable. Owing to extremely lung protective nature of Vitamin B3, this supplement should be used as soon as coughing starts.^74^

#### 3.10.2 Vitamin D

Bovine coronavirus infection in calves in the past has been correlated with the decreased level of vitamin D.^89^ Therefore, inclusion of vitamin D as a nutritional supplement may serve as a potential intervention to combat COVID‐19. Vitamin D is produced by our body in the presence of sunlight. It may reduce the risk of lung infections by inducing cathelicidins and defensins, the antimicrobial peptides, present in airway secretions and expressed by airway epithelium.^90^ These antimicrobial peptides lower rate of viral replication, reduce levels of pro‐inflammatory cytokines and increase the concentrations of anti‐inflammatory cytokines.

Among the several micronutrients, a lack of vitamin D has been associated with an increased chance that COVID‐19 patients may end up in the hospital. According to reports, vitamin D is an important regulator of the renin‐angiotensin system, which the SARS‐CoV‐2 uses to enter the host cell. The many defence mechanisms of the immune system, including the restriction of viral entrance into the host cell, are modulated by vitamin D as well. A cytokine storm is less likely to occur when the host has appropriate amounts of vitamin D due to the decreased release of pro‐inflammatory cytokines, elevated levels of anti‐inflammatory cytokines, and greater secretion of naturally occurring antimicrobial peptides. Additionally, it could have a role in the stimulation of the Th2 immune response and the activation of immune system defences such macrophages. Moreover, vitamin D supplementation in children during early ages could be the reason underlying variability of COVID‐19 infection in children and adults.^2^ In contrast to these findings, numerous investigations have shown no evidence of a connection between high vitamin D levels and a bad prognosis for the illness.^91^

#### 3.10.3 Concerns

Vitamin B3 paradoxically lead to the development of significant hypoxemia.^87^ Doses and timing of vitamin administration needs to be optimized to prevent toxicity.

### 3.11 Probiotics

Probiotics are micro‐organisms that exhibit beneficial effects to the host in improving its innate immune response and providing disease resistance.^92^ Probiotics and prebiotics maintain the intestinal microbiota equilibrium and minimize the risk of secondary infection due to bacterial translocation.^93^ Probiotic strains like *Lactobacillus casei* reduce TLR4 expression hence, reducing the expression of pro‐inflammatory cytokines.^94^ Intranasal inoculation of *Lactobacillus* has shown protective effects against pneumonia in mice.^95^ Therefore, probiotics may prove valuable when administered in combination to antivirals. Intestinal microbial dysbiosis in COVID‐19 patients has been treated with good nutritional diet along with inclusion of probiotics.^93^, ^96^ The role of lipopeptides derived from the probiotics have been established in inactivating spike protein of SARS‐CoV‐2 and ACE2.^97^ The lungs are not sterile and have their own microbiota.^98^ A relationship between gut‐lung axis has suggested that any change or alteration in the microbiome of the gut has implications on the lung microbiome,^99^, ^100^ which may influence the severity of the respiratory conditions in COVID‐19.^101^ COVID‐19 affects mainly old persons, which might be because microbiome dysbiosis occurs more commonly in older ages.^101^, ^102^ Hence, emphasis is being made to enrich gut microbiota through interventional approach like inclusion of probiotics which will eventually help in strengthening lung immunity. However, more organized studies in this area are needed to reach a solid conclusion.

### 3.12 Prebiotics

Prebiotics are dietary compounds which benefit the growth of microbes that produce metabolites capable of modulating host cells; hence, promoting health.^103^ Resistant starches are compounds minimally digested by the host and microbes in the upper gut, thus, become available in the large intestine as microbial‐accessible carbohydrates.^104^ Resistant potato starch (RPS) has shown to increase butyrate, a histone deacetylase inhibitor, which exert immunomodulatory effects by suppressing NF‐κB and pro‐inflammatory cytokines and hence, inhibiting lung inflammation.^105^ It may inhibit ACE2 receptor expression, which is the main gateway for COVID‐19 into epithelial lung cells.^106^ A clinical trial [NCT04342689] using RPS as an effective therapeutic strategy to combat COVID‐19 is going on.

#### 3.12.1 Concerns regarding the use of probiotics and prebiotics

Probiotics and prebiotics do not pose any significant direct health threat. However, their dose needs to be critically optimized along with other measures regarding maintaining purity, safety and authenticity of the probiotic and prebiotic are crucial. These may cause some mild gastrointestinal side effects in some allergic or immunocompromised individuals. Some probiotic strains of *Lactobacillus* may produce histamines which is problematic for individuals having histamine intolerance. More research is needed in this area to explore the mechanism of action of probiotics and prebiotics for proper understanding of their effects on health.

### 3.13 Camelid‐derived nanobodies

Camels produce relatively unique heavy homodimeric chain‐only antibodies IgG, namely nanobodies (Nbs), heavy‐chain antibodies (HcAbs), single‐domain antibodies (sdAbs), or antigen‐specific variable domains (VHH).^107^ They are characterized by high specificity, chemo‐ and thermostability, solubility, reduced sensitivity to steric hindrances, the capacity to assemble multimers to reinforce the binding *in vitro*, and small size (15 kDa), which enable them to detect novel antigenic sites and penetrate tissues deeply.

Nanobodies have promised anti‐inflammatory properties, as demonstrated by the anti‐IL‐6R Nanobody® ALX‐0061 (Ablynx), which is principally designated for the treatment of rheumatoid arthritis. Similar to the widely utilized anti‐IL‐6R monoclonal antibody Tocilizumab (Actemera®), Nanobody ® ALX‐0061 may be used in COVID‐19 to minimize pulmonary inflammation.^108^

## 4 FUTURE PERSPECTIVES

The therapies that lower down lung inflammatory reactions should be highly specific and associated with minimal risks. Hence, vigorous research needs to be done around identification of novel and specific putative or potential biological targets that could help minimize cytokine storm and inflammation. It is advisable to administer antiviral and anti‐inflammatory therapies or drugs early in the COVID‐19 infection because once if the pulmonary inflammation becomes uncontrollable, it might lead to immunosuppression, ARDS, and pneumonia‐causing severe lung injury.^109^

COVID‐19 is a novel contagious disease for which many questions still need to be answered, and future research needs to be conducted. It is still not known whether it is the decline in the number of anti‐inflammatory responses or the elevated production of pro‐inflammatory mediators/responses which adds to the severity of COVID‐19.^110^ Moreover, further research needs to be done to explore the effects of SARS‐CoV‐2 in the lung at the cellular and molecular level so that more effective options for treatment could be adopted.

Vaccination and herd immunity are going to be effective strategies in the long run, but many questions relating to vaccines remain unanswered. The issues pertaining to vaccination include storage, distribution and availability in huge numbers, duration for which it provides immunity, and efficacy against new variants of coronavirus that need to be addressed. The research in this field is hampered by mutations in the viral genome.^111^, ^112^ The virus is evolving very rapidly, and the genetic diversity of the SARS‐CoV‐2 poses challenges to identify a specific target.^113^, ^114^ Overall, a systematic approach is needed to understand the mechanisms and the factors involved in the pathogenesis of SARS‐CoV‐2 infection leading to subsequent pneumonia and lung cytokine storms. Further, identifying a suitable therapeutic strategy or a combination of strategies will provide timely treatment of this destructive disease.^115^

Considering a significant death rate, only a few COVID‐19 therapies have been studied in severely infected patients with COVID‐19.^116^, ^117^ Because of this, it is quite challenging to determine the relative merits of the many treatment approaches that are now accessible. To analyze the effects of these and other newer medications in severely infected patients with COVID‐19, comprehensive studies are indeed recommended. In this regard, superior trial designs and meticulous selection criteria are required in the future.^118^ It is crucial to avoid treating all individuals with severe COVID‐19 in the same manner. Not all these individuals exhibit the distinctive markers, which include significant endothelial dysfunction, hyperinflammatory conditions, and profound immune system changes that encourage the development of various levels of organ failure. The fact that this triad—hyperinflammation, immunological dysregulation, and endothelial dysfunction—occurs in sepsis as well as COVID‐19 would support the idea that severe COVID‐19 is a kind of viral sepsis. According to distinct phenotypes, these modifications enable the categorization of critically infected patients with SARS‐CoV‐2.^119^, ^120^, ^121^ Recently, a machine‐learning approach has been used to identify two characteristics in a single‐center analysis of severely infected patients with COVID‐19. One of the characteristics is hyperinflammation, which is characterized by enhanced pro‐inflammatory cytokines and greater rates of complications and morbidities. The other distinguishing feature is hypo‐inflammation. Interestingly, corticosteroid treatment was only linked with a decreased 28‐day death rate in individuals with the hyperinflammatory phenotype.^122^ Such endotypes encompass biological and clinical traits and may serve as precise targets for more carefully choosing medicines based on a patient's unique medical needs.^118^ However, there is still an urgent need to pinpoint the effective drugs and their drug targets with in vitro and in vivo experiments.

Recent findings from clinical investigations have shown the possibility of treating COVID‐19 using a number of effective and dependable therapeutic approaches, including antiviral medications, mAbs, convalescent plasma therapy, etc. Despite several developments in the creation of medications that specifically target the viral agent, various treatment approaches that trigger a potent immune response and T‐cell‐driven cytokines have been identified. These tactics also include improving the T‐cell, Th1, and B‐cell responses that are unique to the virus, raising T‐cell numbers, overcoming T‐cell depletion and perturbations, and lowering inflammation.^123^ Adoptive T‐cell transfer (ACT) has been shown in prior research on pathological conditions, particularly viral infections, to be a successful method for increasing T‐cell numbers and reversing T‐cell exhaustion. The number of T cells needed to control COVID‐19 may be increased by using virus‐specific T cells and transmission by ACT.^123^

## 5 CONCLUSIONS

The COVID‐19 problem is cumbersome as the infection can affect even the vaccinated populations and cytokine storm has been observed in certain cases following COVID‐19 vaccination highlighting the immune dysregulation. It has been observed during SARS‐CoV‐2 infection that some factors in the host trigger a heightened immune response to eliminate the virus that eventually might lead to lung inflammation or lung tissue damage.^124^ Therefore, normalizing lung inflammation in COVID‐19 could be a game changer and may save lives by lessening the chances of developing fatal ARDS and pneumonia. However, the benefits of using immunomodulatory approaches should outweigh the risks associated with these strategies as discussed in the present review. Research should come up with therapies that do not pose potent risks and are effective and safe. Dietary changes, incorporation of probiotics and probiotics, and supplementation of vitamins in the diet should be emphasized as these strategies seem to be promising in strengthening the immune system.^125^ However, ongoing questions, such as how much of a dosage to take, when to take it, and for which patients these approaches are most likely to help, are all things that will be looked at more in the future.

## AUTHOR CONTRIBUTIONS

Geetika Verma: Conceptualization (lead); writing – original draft (lead); writing – review and editing (equal). Manish Dhawan: Software (lead); writing – review and editing (equal). AbdulRahman A. Saied: Writing – review and editing (supporting). Geetika Kaur: Writing – review and editing (supporting). Reetesh Kumar: Writing – review and editing (supporting). Talha Bin Emran: Writing – review & editing (equal).
